# A Case of Spontaneous Pneumomediastinum Following Ecstasy and Marijuana Use

**DOI:** 10.7759/cureus.15871

**Published:** 2021-06-23

**Authors:** Mishouri Paul, Prodip Paul, Dipon Dey, Amit Bhardwaj, Koushik Paul

**Affiliations:** 1 Medicine, Interfaith Medical Center, New York City, USA; 2 Internal Medicine, Geisinger Community Medical Center, Scranton, USA; 3 Epidemiology and Public Health, ZWH Medical Care PC, Queens, USA; 4 Clinical Pathology, Community Based Medical College Hospital, Mymensingh, BGD

**Keywords:** pneumomediastinum, subcutaneous emphysema, ecstasy, marijuana, boerhaave's syndrome

## Abstract

Spontaneous pneumomediastinum (SPM) is a benign and self-limiting condition more commonly seen in young adults. Radiology imaging of the chest, including X-ray or CT scan, is the gold standard for diagnosis. Ecstasy, also known as 3,4-methylenedioxymethamphetamine (MDMA) is a synthetic amphetamine derivative widely abused for an increased sense of well-being and euphoria. Marijuana is also abused for recreational purposes. SPM has been reported after both Ecstasy and marijuana use. SPM after these illicit drugs abuse usually has a benign and self-limiting course with supportive management. However, it is always important to rule out serious associated conditions like esophageal perforation. Here, we present a 22-year-old male who developed SPM after Ecstasy ingestion and marijuana inhalation.

## Introduction

Spontaneous pneumomediastinum (SPM) is a rare condition with an incidence estimated between 0.001% and 0.01% in adult patients. It is commonly seen in young males with chest pain, shortness of breath, and subcutaneous emphysema [1]. Exacerbation of asthma, tobacco, corticosteroids, vomiting, cough, physical exercise, labor, and abuse of recreational drugs such as cocaine, marijuana, and Ecstasy have been reported to trigger pneumomediastinum [2,3]. Excessive positive airway pressure causes overinflation and rupture of alveoli [2]. Escape of air through the perivascular alveoli into the mediastinum along a pressure gradient, and thereafter to the fascial planes of the neck is responsible for SPM [4].

Marijuana is the most commonly used psychotropic drug in the United States, after alcohol. In 2018, more than 11.8 million young adults reported using marijuana in the past year. A case report has been published on how performing Valsalva maneuvers during marijuana smoking causes pneumomediastinum [5]. SPM in marijuana users is believed to result from either tearing the esophagus due to cyclic vomiting or microperforation of the esophagus [6] or from barotrauma during breathing maneuvers [5,7].

Ecstasy is an illegal street drug widely abused by adolescents and young adults for recreational purposes [2,8]. The active ingredient of Ecstasy is 3,4-methylenedioxymethamphetamine (MDMA). It induces a state of increased alertness and euphoria secondary to its stimulant activity in the central nervous system. Due to its chemical similarity related to the psychostimulant methamphetamine and the hallucinogen mescaline, the drug produces stimulating and mild hallucinogenic effects [9]. Both Ecstasy ingestion and inhalation are associated with SPM [2,10]. Although there are no reports of mortality directly related to Ecstasy-associated SPM, ecstasy ingestion may lead to significant and potentially life-threatening complications including hyperthermia, cardiac arrhythmias, seizures, fulminant hepatic toxicity, and acute renal failure [2]. Therefore, it is important to monitor the patient during the brief period of hospitalization.

Once more, fatal condition of esophageal perforation is ruled out, Ecstasy and marijuana-related pneumomediastinum are managed with conservative measures [4,7]. Here, we present a 22-year-old male who developed SPM with subcutaneous emphysema after smoking marijuana and Ecstasy ingestion.

## Case presentation

**Figure 1:**
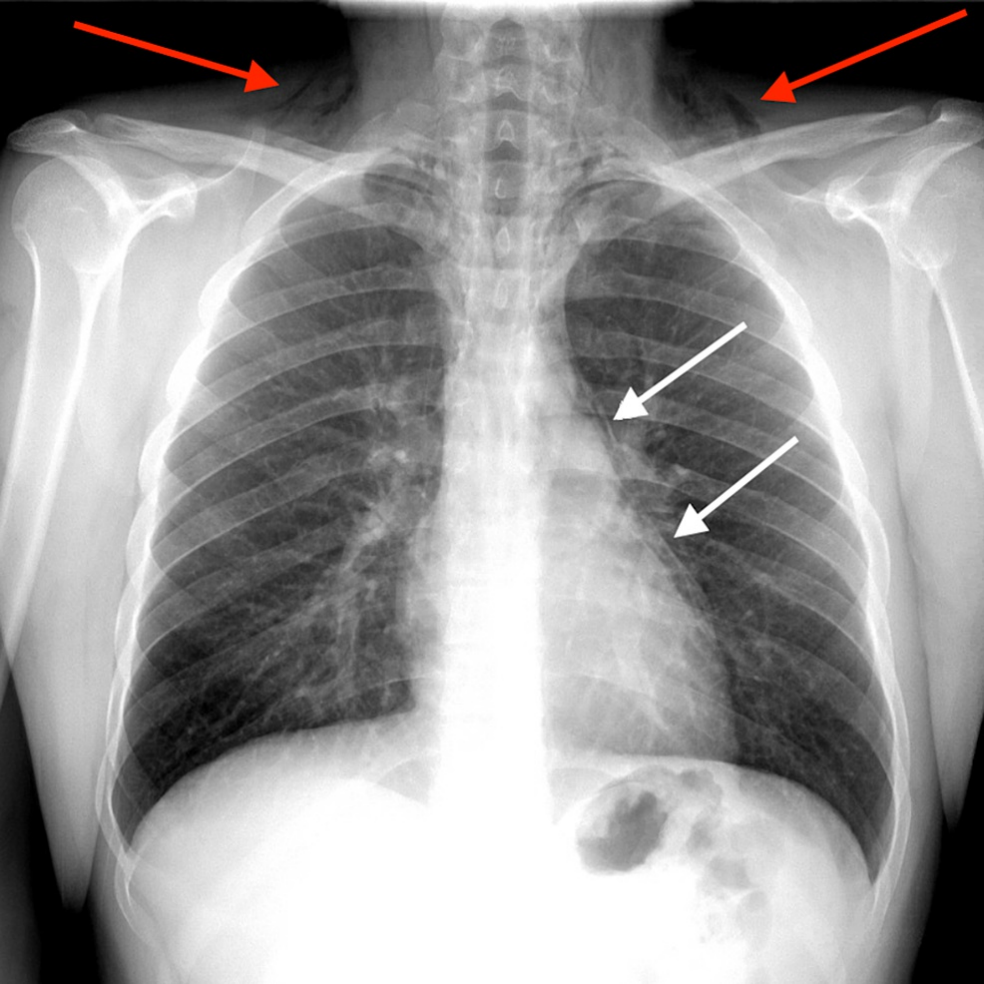
Chest x-ray showing subcutaneous emphysema (red arrows) and pneumomediastinum (white arrows)

A 22-year-old male with a past medical history of bronchial asthma presented to the emergency department with complaints of sudden onset neck and chest pain. The patient stated that around midnight which was about 20 hours prior to arrival to ED, he ingested ecstasy and smoked marijuana. The patient was awake and active the whole night and he was doing well until morning. Then he went to take a shower when he had an episode of the projectile and non-bloody vomiting. The patient then noticed some changes in his voice. The patient had another episode of projectile vomiting. Since then, the patient complained of pain in the neck and chest. The patient denies any shortness of breath, cough, nausea, or any further episodes of vomiting. The patient also denies any fever or chills. The patient had a history of asthma that had been well controlled. The patient reported marijuana smoking at least once a week for the past several years. On examination, the patient was in no acute distress, other than tachycardia hemodynamically stable, saturating well on room air. On palpitation, crepitus was noted over the lower part of the neck and upper chest wall. Chest x-ray shows pneumomediastinum and subcutaneous emphysema (Figure 1).

**Figure 2:**
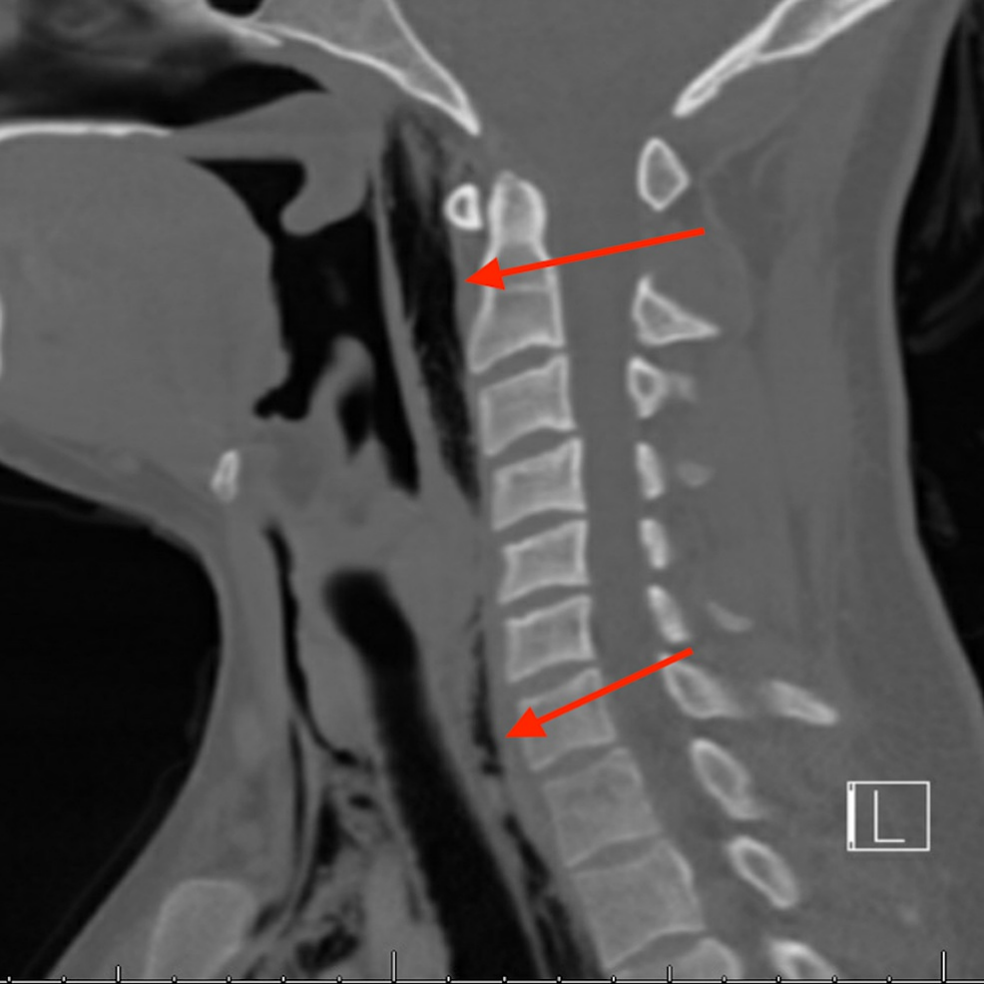
CT scan of the neck with intravenous contrast (sagittal section) showing gas tracking into the retropharyngeal space (red arrows) L - left

**Figure 3:**
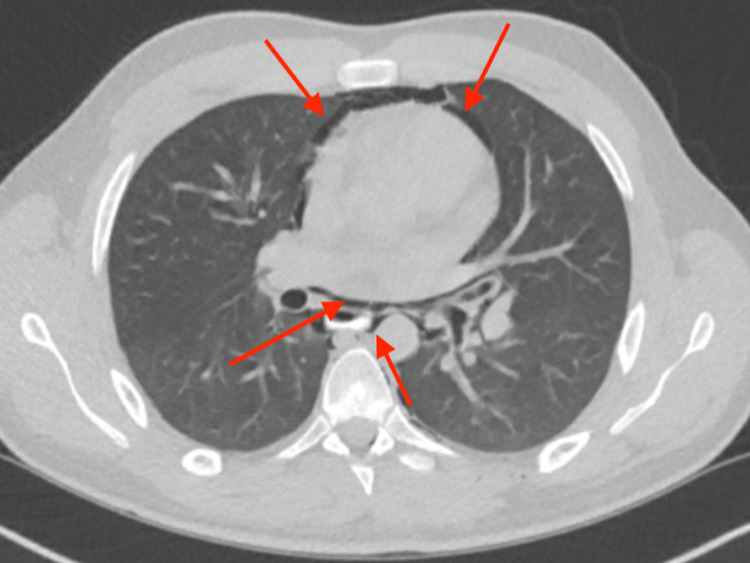
Axial CT scan showing curvilinear lucencies along mediastinal structures compatible with pneumomediastinum (red arrows)

CT scan of the neck with intravenous contrast showed extensive gas involving the neck. Involved spaces included submandibular space, sublingual space, perivertebral space, and retropharyngeal space (Figure 2). The cardiothoracic surgeon evaluated the patient. The patient also had a CT scan of the chest with oral contrast, and it showed extensive pneumomediastinum (Figure 3). Esophagogram was performed as well to rule out Boerhaave’s syndrome. Both CT scan of the chest with oral contrast and esophagogram did not reveal any esophageal leak.

**Figure 4:**
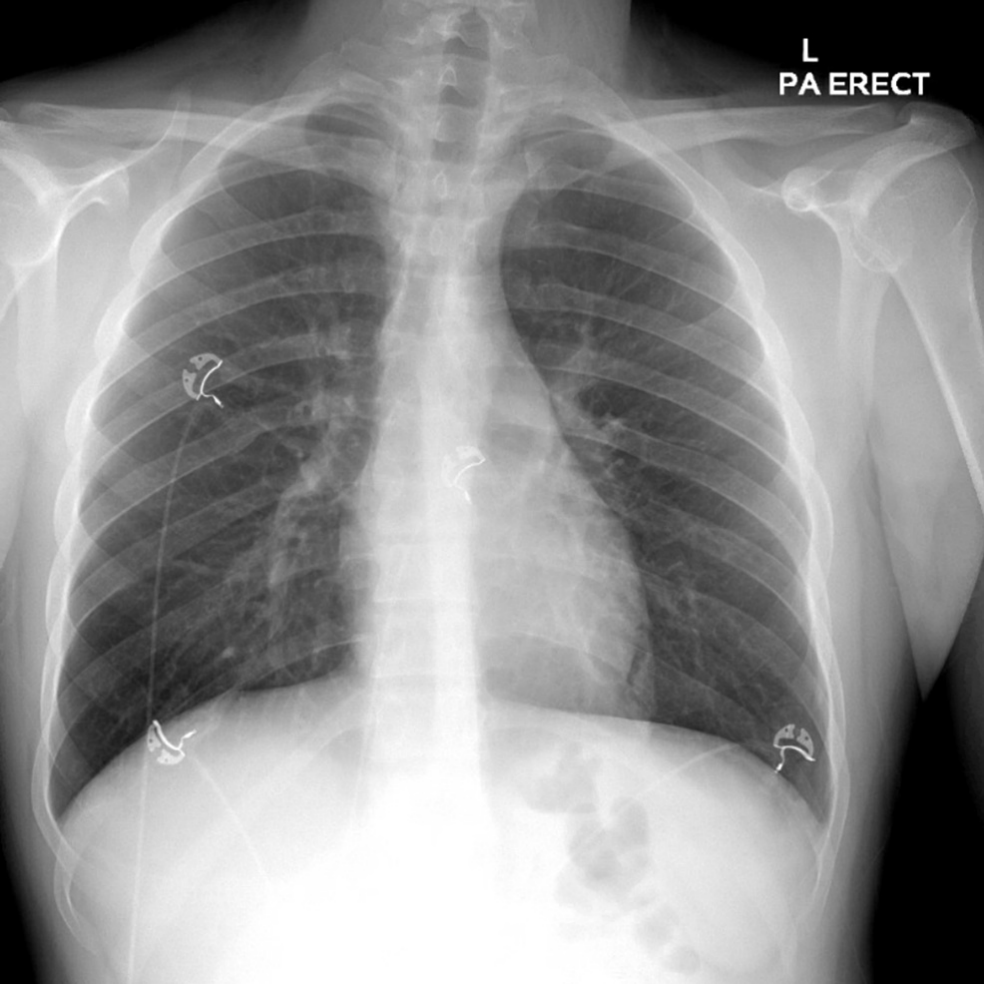
Chest x-ray showing improvement in pneumomediastinum and subcutaneous emphysema L - left; PA - posteroanterior

The patient was admitted for monitoring and conservative management. The urine drug screen was positive for methamphetamine and cannabinoid. The otolaryngologist and gastroenterologist also evaluated the patient. Boerhaave’s syndrome was ruled out and SPM was thought to be related to Ecstasy and marijuana use. Repeat serial chest imaging showed continued improvement in pneumomediastinum as well as decreasing gas within the overlying cervical soft tissues (Figure 4).

The patient was discharged in stable condition on the fourth day of hospitalization.

## Discussion

SPM and subcutaneous emphysema are more common phenomena after inhaled drugs than after ingested substances. Inhalation of drugs such as cocaine, marijuana, or Ecstasy followed by SPM, and subcutaneous emphysema can be explained by the barotrauma induced by the ritualistic Valsalva maneuver [11].

Inhalational use of marijuana has been associated with respiratory symptoms such as chronic cough, sputum production, dyspnea, and hoarseness [12], and has recently been described as a possible risk factor for chronic bullous lung disease and secondary pneumothorax [13]. A recent retrospective review revealed that SPM is associated with a history of inhaled marijuana use [7]. Although coughing is generally the most common precipitating factor for causing SPM, smoking techniques and marijuana-induced vomiting play a key role in causing SPM in marijuana users [7]. Moreover, vomiting was reported as the suspected mechanism of pneumomediastinum after Ecstasy ingestion [14,15]. In this case, the patient had two episodes of projectile vomiting after Ecstasy ingestion and marijuana inhalation, and immediately after the second episode of vomiting the patient developed neck and chest pain.

Patients with Ecstasy-related pneumomediastinum present with pleuritic chest pain with radiation to the neck [16] or back [17], neck swelling corresponding to underlying dysphonia [14], dysphagia [17], subcutaneous emphysema [18], and odynophagia [18]. The time of presentation varies as patients present several hours to days after the consumption of the substance. Most common examination findings include subcutaneous emphysema, tachycardia, and Hamman’s sign [4]. In this case, the patient presented with complaints of neck and chest pain, and the symptoms developed approximately nine hours after ecstasy ingestion and marijuana inhalation. On initial examination, the patient was noted to have tachycardia, and there was crepitus over the neck and chest wall indicating subcutaneous emphysema.

The recommended imaging modality in SPM is a chest radiograph [1]. Patients with pneumomediastinum or pneumopericardium should be arranged for an immediate contrast swallow to rule out esophageal rupture [18]. Pneumomediastinum is a benign and self-limiting condition. After excluding esophageal rupture, supportive management should be given with gradual reintroduction of fluids and food by mouth [15]. In this case, after initial evaluation, the patient had a chest x-ray, which revealed pneumomediastinum. Following the diagnosis of pneumomediastinum, esophagogram was performed and esophageal rupture was ruled out. The patient was then managed conservatively with gradual improvement of pneumomediastinum and subcutaneous emphysema.

## Conclusions

SPM has been reported with both Ecstasy and marijuana use. History should be obtained regarding illicit drug abuse when dealing with pneumomediastinum especially in adolescent and young patients. Once the more fatal conditions of esophageal rupture and pneumothorax have been ruled out, Ecstasy and marijuana-induced SPM are managed conservatively with a self-limiting course.
